# A virtual simulation approach to assess the effect of trocar-site placement and scar characteristics on the abdominal wall biomechanics

**DOI:** 10.1038/s41598-024-54119-4

**Published:** 2024-02-13

**Authors:** Lluís Tuset, Manuel López-Cano, Gerard Fortuny, Josep M. López, Joan Herrero, Dolors Puigjaner

**Affiliations:** 1 Departament d’Enginyeria Informàtica i Matemàtiques, Universitat Rovira i Virgili, Av. Països Catalans 26, Tarragona, Catalunya Spain; 2 Abdominal Wall Surgery Unit, Department of General Surgery, Hospital Universitari Vall d’Hebron, Universitat Autònoma de Barcelona, Barcelona, Spain; 3 Departament d’Enginyeria Química, Universitat Rovira i Virgili, Av. Països Catalans 26, Tarragona, Catalunya Spain

**Keywords:** Biomedical engineering, Colorectal surgery

## Abstract

Analyses of registries and medical imaging suggest that laparoscopic surgery may be penalized with a high incidence of trocar-site hernias (TSH). In addition to trocar diameter, the location of the surgical wound (SW) may affect TSH incidence. The intra-abdominal pressure (IAP) exerted on the abdominal wall (AW) might also influence the appearance of TSH. In the present study, we used finite element (FE) simulations to predict the influence of trocar location and SW characteristics (stiffness) on the mechanical behavior of the AW subject to an IAP. Two models of laparoscopy patterns on the AW, with trocars in the 5–12 mm range, were generated. FE simulations for IAP values within the 4 kPa–20 kPa range were carried out using the Code Aster open-source software. Different stiffness levels of the SW tissue were considered. We found that midline-located surgical wounds barely deformed, even though they moved outwards along with the regular LA tissue. Laterally located SWs hardly changed their location but they experienced significant variations in their volume and shape. The amount of deformation of lateral SWs was found to strongly depend on their stiffness. Trocar incisions placed in a LA with non-diastatic dimensions do not compromise its mechanical integrity. The more lateral the trocars are placed, the greater is their deformation, regardless of their size. Thus, to prevent TSH it might be advisable to close lateral trocars with a suture, or even use a prosthetic reinforcement depending on the patient's risk factors (e.g., obesity).

## Introduction

The prevalence of trocar-site hernias (TSH) is not clear^1^. Analysis of registries suggests a high incidence of TSH^2^ and that, depending on their location, reparation of these hernias can be complex^3^. Moreover, the recently updated guidelines for closure of abdominal wall incisions^4^ provide no support to the notion that the fascial closure at the trocar site can benefit TSH prevention. These guidelines also mention that there is no evidence supporting the best trocar location and they recommend suturing the fascial defect for trocar sites of 10 mm or larger, and for trocars located at the umbilical site^4^.

Finite element (FE) simulations may strengthen our understanding of how surgical wounds (SWs) alter the mechanical behavior of the abdominal wall (AW)^5,6^. Our key assumption is that the response of the AW to intra-abdominal pressure (IAP) sometime after surgery will depend on the mechanical properties of the tissue regrown at the surgical sites, that is, the tissue conforming the SWs, and the laparoscopy pattern used in the surgery. The resilience of the AW to an applied IAP value, *P*_*a*_*,* is mostly due to the stiffness of its tissues, which are of muscular or tendinous nature. Both types of tissue are characterized by the presence of fibers in their architecture^7^. The new SW tissue might be weak and, from a mechanical point of view, softer than expected^8^. On the other hand, the regrown tissue in the SW might become scarred^9,10^ and exceedingly stiff^11^.

In silico analysis could help to virtually simulate the influence of trocar location or trocar scar characteristics on the mechanical behavior of the AW. However, to the best of our knowledge, there is no study on how the SW characteristics, or its location alter the mechanical response of the AW. The aim of this work is to use FE simulations to predict AW deformation as a function of the stiffness and location of the SWs (mimicking trocar-site placement), and the level of IAP applied.

## Models and methods

### Geometry model

**Figure 1:**
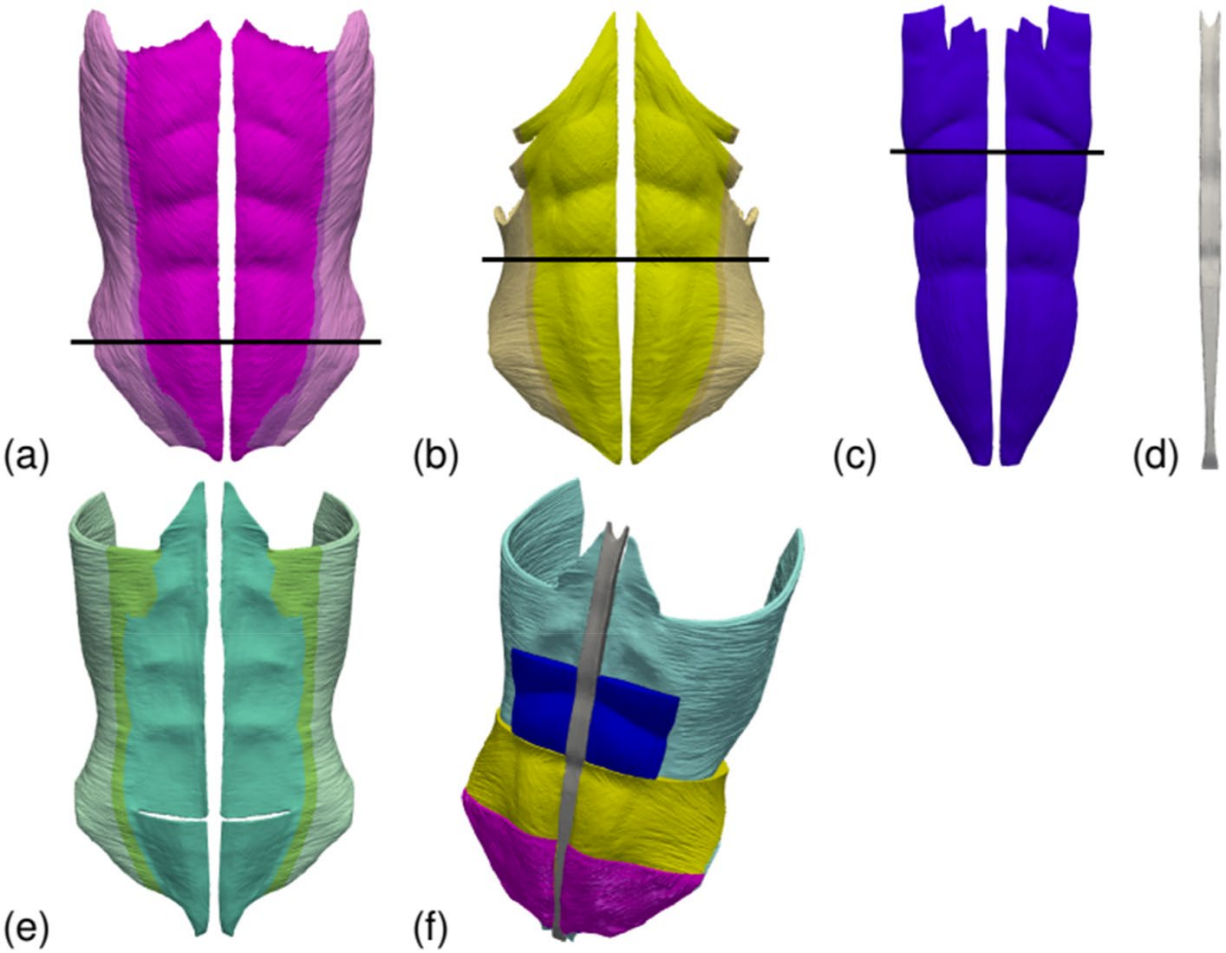
Geometry model of the abdominal wall. The elements involved in the model are: (**a**) right and left external oblique muscles (EO), (**b**) right and left internal oblique muscles (IO), (**c**) right and left rectus abdominis muscles (RA), (**d**) linea alba (LA) and (**e**) right and left transverse abdominis muscles (TR). (**f**) View of the whole model. Note that as the involved muscles are superimposed only those muscle regions below black lines in parts (**a**–**c**) are displayed. In parts (**a**,**b**,**e**) the different regions (regular, intermediate, and aponeurotic muscle tissue) are denoted with increasingly saturated color tones.

The present geometry model, illustrated in Fig. 1, is similar to the one used in our previous work^5^. It consists of external oblique (EO), internal oblique (IO), rectus abdominis (RA) and transverse abdominis (TR) muscles, as well as the linea alba (LA), which was considered with standard dimensions of 2 cm (i.e., without rectus diastasis)^12^. Notwithstanding, a novelty in the present geometry model is that the aponeurosis of the TR, EO and IO muscles is considered as a distinct tissue, having mechanical properties different from those of the regular muscle (see Fig. 1).

**Figure 2:**
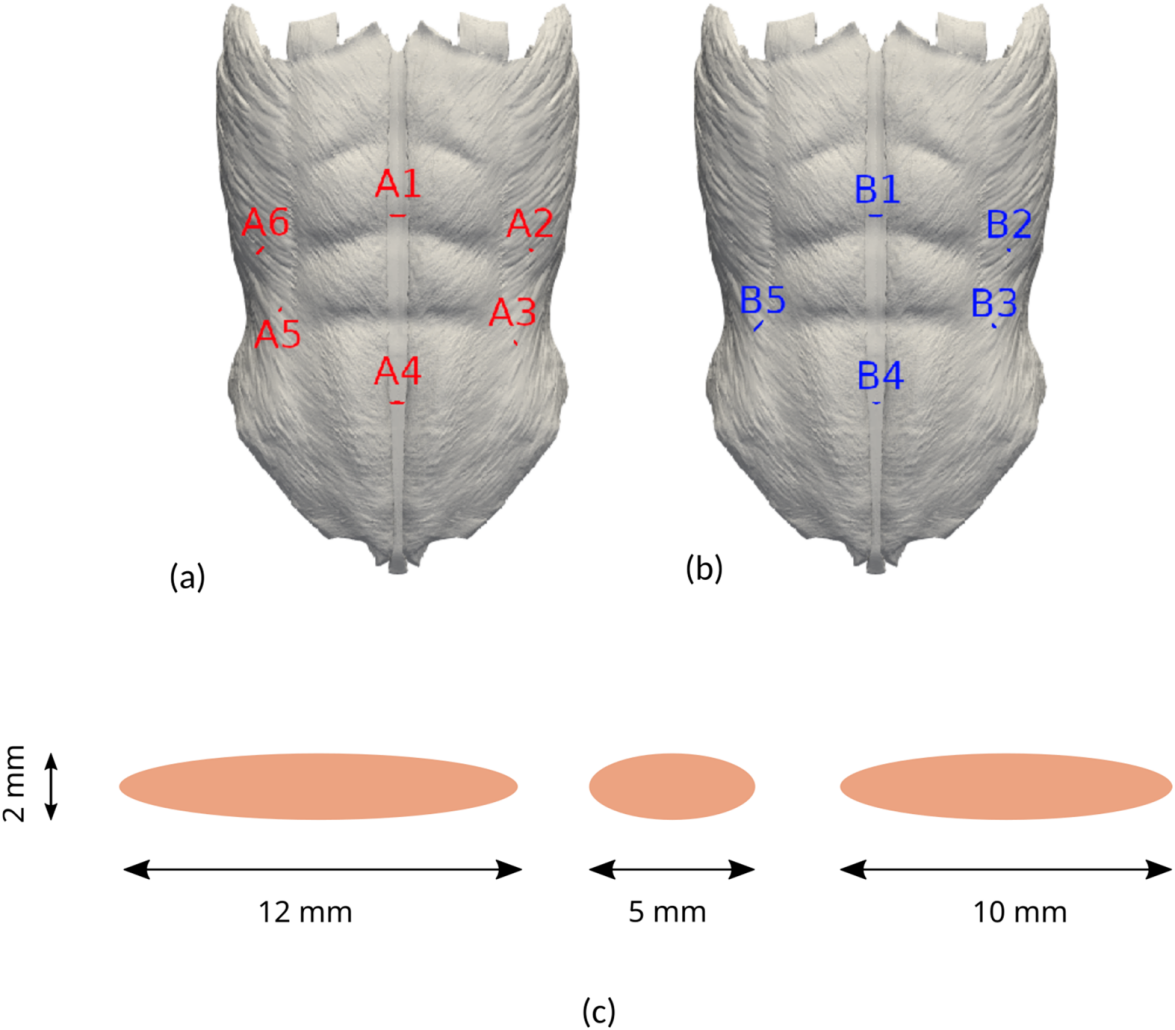
Distribution of the incisions on the AW for the two laparoscopy patterns studied in this work. (**a**) Laparoscopy model A, with three 5-mm long and three 12-mm long incisions. (**b**) Laparoscopy model B, with one 10-mm long, one 12-mm long and three 5-mm long surgical wounds (see Table 1 for details on the length, surface area and volume of each laparoscopic SW). (**c**) Sketch of the elliptic profile for the three geometry models of laparoscopy surgical wounds assumed in the present study.

We investigated two laparoscopy patterns, denoted as models A and B (see Fig. 2a,b). We considered surgical wounds after surgery, i.e., assuming all trocar-sites closed. SWs were modeled as elliptic cylinders orthogonal to the outer AW surface (see Fig. 2c) with the ellipse major axis representing the trocar diameter, and the minor axis set to 2 mm in all cases. We will henceforth refer to the length and width of a laparoscopy SW instead of to the ellipse axes.

Length, surface area and volume of each SW geometry are summarized in Table 1. Model A (Fig. 2a) comprises six surgical wounds. Two 12-mm long incisions are located on the LA (A1, A4), two 5-mm long incisions are located on the left lateral region (A2, A3), and two more SWs, respectively 5- and 12-mm long, are on the right lateral region (A5, A6). Model B (Fig. 2b) consists of five surgical wounds. Two incisions, a 10-mm long supraumbilically placed (B1) and a 5-mm long infraumbilically placed (B4), are embedded in the LA. Two 5-mm long incisions are located on the left lateral region (B2, B3) and a 12-mm long SW is placed on the right lateral region (B5).

**Table 1:** Dimensions of every SW in the present laparoscopy trocar models.

	length (mm)	surface area (cm^2^)	volume (cm^3^)		length (mm)	surface area (cm^2^)	volume (cm^3^)
A1	12	3.31	0.217	B1	10	2.78	0.179
A2	5	3.00	0.190	B2	5	3.00	0.190
A3	5	2.27	0.142	B3	5	2.18	0.135
A4	12	3.48	0.231	B4	5	1.67	0.100
A5	5	2.16	0.134	B5	12	4.80	0.332
A6	12	6.78	0.477				

### Material properties

**Figure 3:**
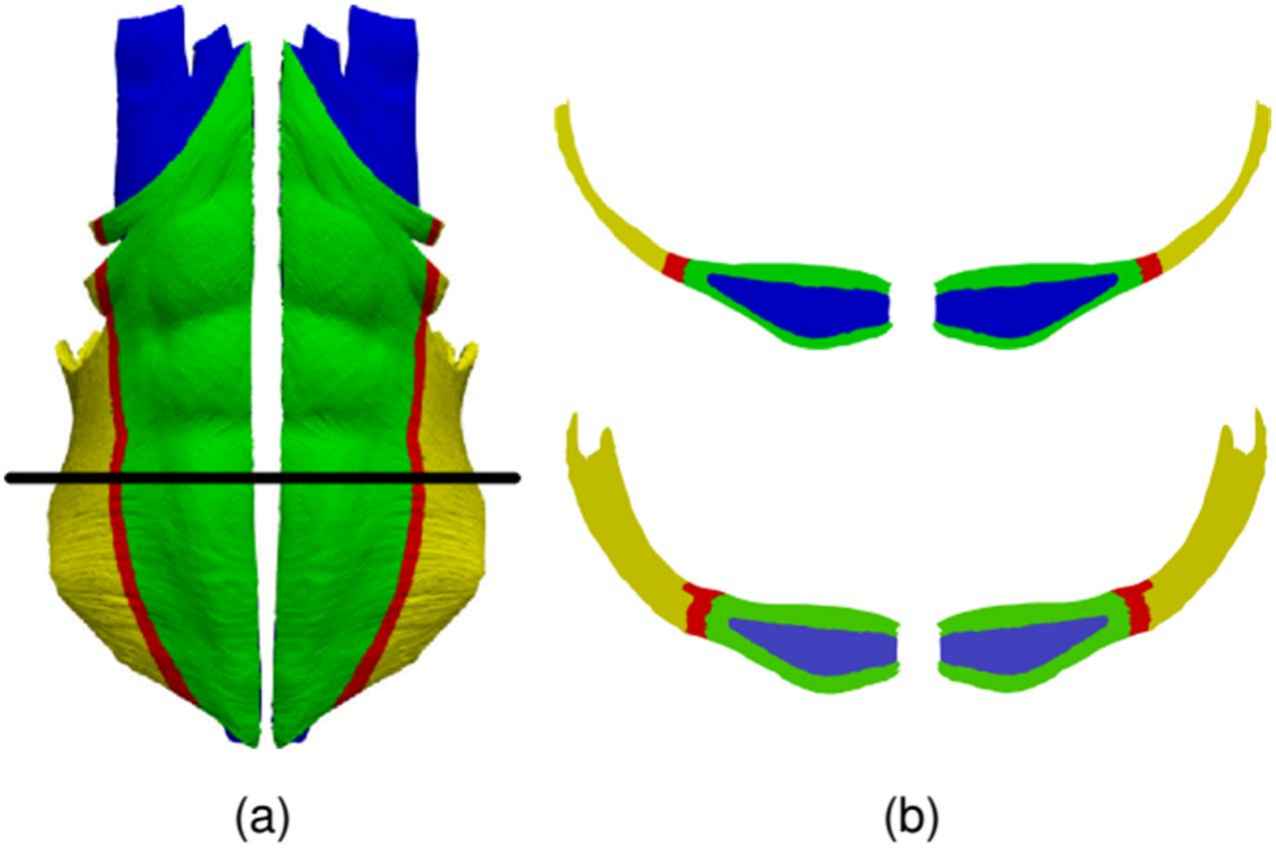
Illustration of the transition between the regular muscle tissue (yellow), intermediate tissue (red) and aponeurotic tissue (green). (**a**) Frontal view of the left and right IO muscles that surround most of the RA muscles (blue). (**b**) Axial slices taken at the height denoted by the horizontal black line in part (**a**); the upper plot shows only the IO and RA tissues whereas the lower plot shows also the EO and TR muscle tissues for the sake of completeness.

Following our previous work^5^ we assumed a linear elastic behavior of the AW tissues,

1 $$\sigma = E\varepsilon$$

where **σ** is the stress tensor, **ε** is the strain tensor, accounting for the relative deformation of tissues, and E is the elastic modulus. The specific E values for each tissue, together with the bibliographic source, are listed in Table 2. A value of *ν* = 0.49 was assumed for the Poisson's ratio of muscular tissues. The aponeurosis of the TR, EO and IO muscles forms the so-called rectus sheath^13,14^. The tissue in the rectus sheath is more fibrous (higher E) than regular muscular tissue. Figure 3 illustrates the definition of the three regions (regular muscle, rectus sheath, and a thin intermediate transition region). In the transition region, we assumed an E value halfway the values for rectus sheath and regular muscle.

**Table 2:** Elastic modulus (*E*) assumed for the different tissues in our AW model.

Tissue		*E* (MPa)	References
RA		0.52	Cardoso^28^
LA		72	Cooney et al.^29^
EO	Regular muscle	1	Cardoso^28^
	Transition region	3.3	
	Rectus sheath	5.6	Ben Abdelounis et al.^30^
IO	Regular muscle	0.65	Cardoso^28^
	Transition region	3.1	
	Rectus sheath	5.6	Ben Abdelounis et al.^30^
TR	Regular muscle	1.03	Cardoso^28^
	Transition region	3.3	
	Rectus sheath	5.6	Ben Abdelounis et al.^30^

*RA* Rectus Abdominis, *LA* Linea Alba, *EO* Internal Oblique, *IO* Internal Oblique, *TR* Transverse Abdominis.

A wide variability in the mechanical properties of wounds can be found in the literature. For example, Ibrahim et al.^15^ reported E values of a few kPa for uninjured human skin, whereas Samartsev et al.^16^ obtained elastic moduli of a few GPa in their experiments with sutured samples. In the current study, we performed simulations within a wide range of E values for the SWs, namely 0.1 MPa ≤ E_w_ ≤ 10,000 MPa. The Poisson ratio for SW tissue was set to *ν*_w_ = 0.40^16^.

### Numerical simulation

**Figure 4:**
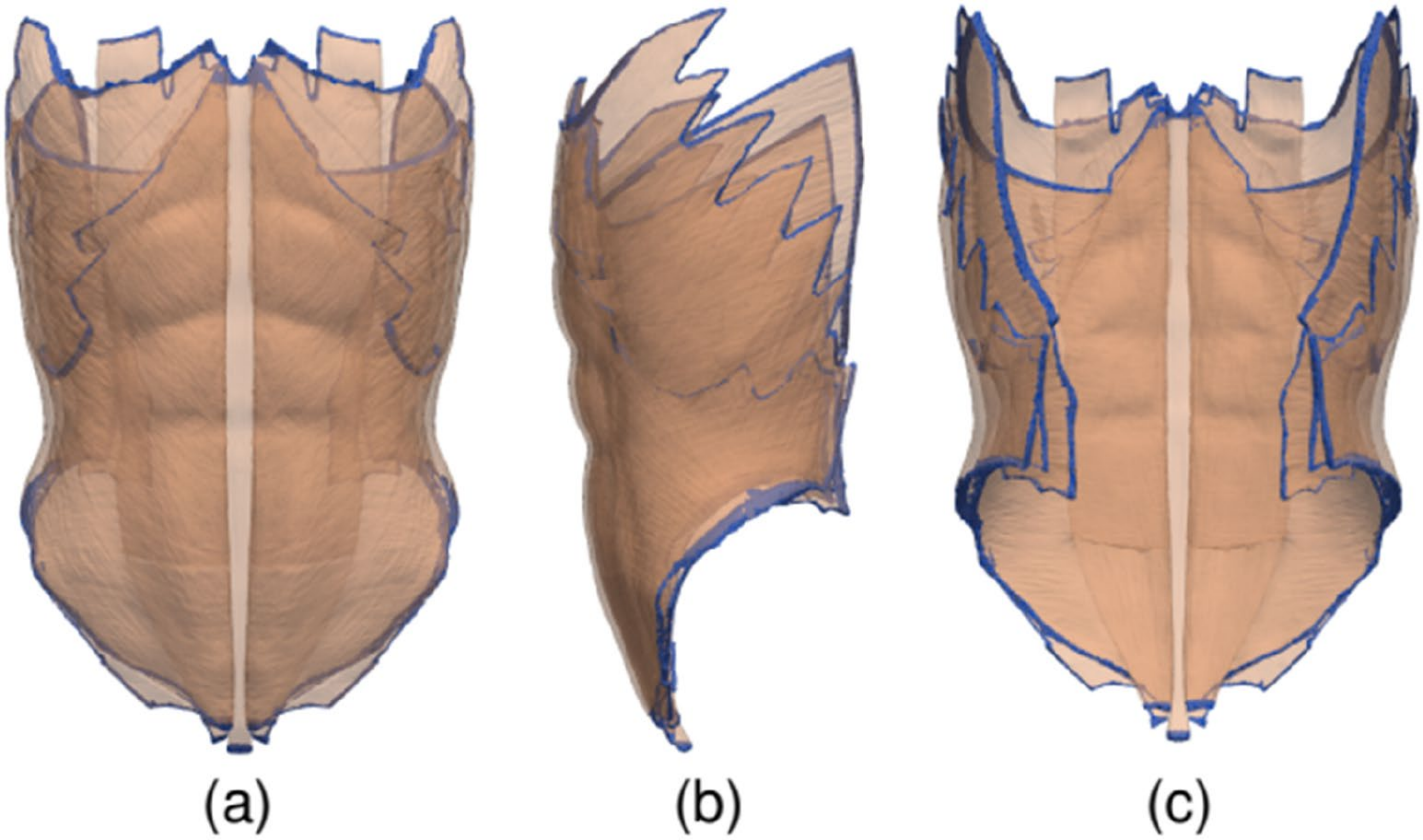
Views of the AW geometry with a dark blue color denoting areas where the fixed boundary condition is applied. (**a**) Front view. (**b**) Side view. (**c**) Rear view. Note that the surfaces of the different geometry elements (muscles) have been made translucent to enhance the visibility of fixed-boundary areas.

The current simulations were performed using the Code Aster open-source FE software^17^. As illustrated in Fig. 4, fixed (zero deformation) boundary conditions were prescribed at the edge of the abdominal wall, where the muscles would attach to the bones. The IAP was uniformly applied to the AW inner surface. For each geometry, we performed simulations for five uniformly distributed values pf *P*_a_ between 4 and 20 kPa, a normal IAP range during typical activities of daily living^18^. In each simulation, the resulting deformations of the geometry were computed and analyzed.

**Figure 5:**
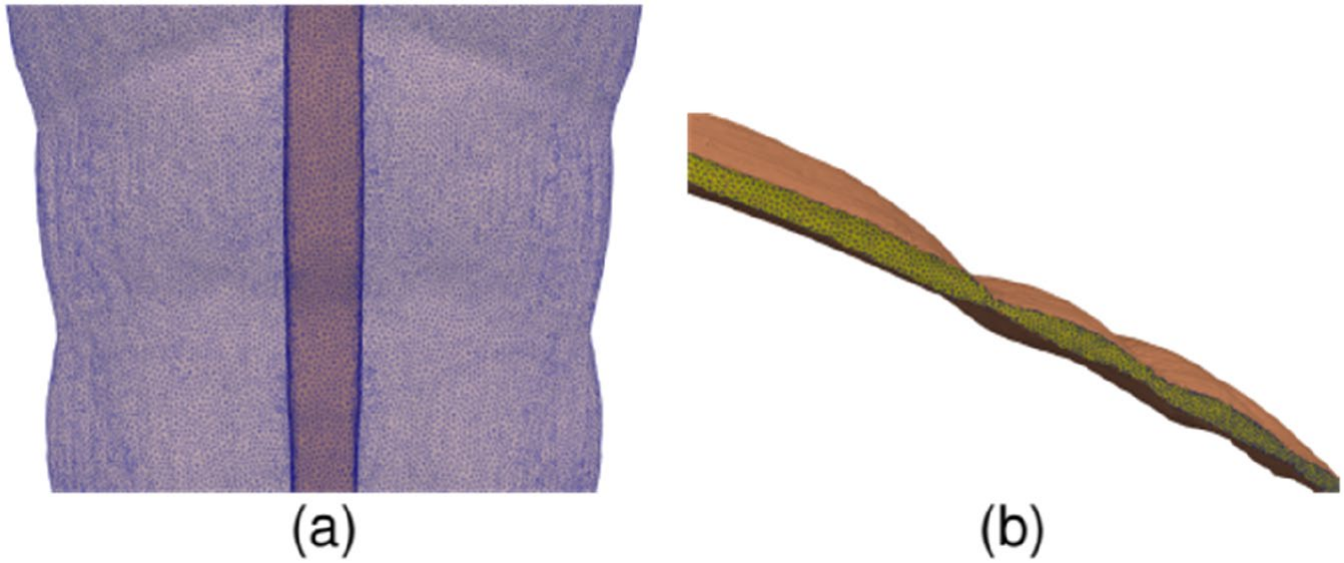
(**a**) Detail of the computational mesh on a sector of the RA muscles (pink) and LA (brown) frontal surfaces; the edges of the surface-mesh triangles are denoted with blue lines. (**b**) Side view of a cross-section of the left RA mesh showing the lateral surface (yellow, with blue triangle edges) that is shared with the LA mesh.

The geometry model was generated by refinement of the raw model available in the BodyParts3D database for anatomy, which in turn was generated from CT images of an adult man^19^. In this refinement process, internally consistent, high quality triangular surface meshes were generated for each of the AW components in our model. Subsequently, the corresponding volume meshes, consisting of linear tetrahedra, were constructed. Table 3 shows the dimensions of the three computational meshes used in the present study, i.e., the mesh for the base case (no-trocar) and the respective meshes for the trocar patterns A and B. Special care was taken to ensure the homogeneity of the computational meshes. That is, the values of the (mesh wide) mean triangle surface area and mean tetrahedron volume, listed in Table 3, are characteristic of most of the mesh elements. A exception to this rule is found in the particularly refined meshes that were generated for the trocar elements (see Table 3). These trocar meshes replaced the corresponding portions of the base mesh at the trocar sites and the mesh elements in the surrounding regions were correspondingly adapted to ensure continuity and smoothness of the resulting computational mesh. As an example, Fig. 5a shows a section of the surface mesh of the RA muscles and LA whereas Fig. 5b provides a detailed view of the mesh on the internal surface separating, and common to, the left RA and LA volumes.

**Table 3:** Main features of the computational meshes used in the present FE simulations.

	Base case (no trocars)	Model A	Model B
Nodes	714 816	748 607	746 629
Surface mesh triangles	486 620	486 998	486 858
Tetrahedrons	3 495 765	3 712 302	3 701 001
Average triangle area (mm^2^)	0.811	0.811	0.811
Average tetrahedron volume (mm^3^)	0.698	0.658	0.660
Trocar average triangle area (mm^2^)	–	0.235	0.234
Trocar average tetrahedron volume (mm^3^)	–	0.086	0.082

In the three models, the internal total volume is 2441 cm^3^ and the external surface area (volume external boundaries) is 0.3947 m^2^. The number of triangles and their properties refer only to the external surfaces, i.e., those that define the exterior boundary of a volume.

### Characterization of the deformation patterns of incisions

For each laparoscopy pattern (A and B), we first performed a reference case simulation in which E_w_ values for every SW were set to the average E of the muscles surrounding it (that is, SWs were considered to be healed). Then we performed a set of simulations in which one SW at a time was softened to E_w_ = 0.1 MPa. Since the predicted displacements of each computational node are available in the FE simulation output, we computed the corresponding changes in surface area and volume of the deformed SWs by means of numerical integration along the mesh surface elements (triangles) and volume elements (tetrahedra), respectively.

There are two main effects in the deformation of a SW: (i) an undulation of its outer surface, and (ii) a reduction in its length (depth) in the direction normal to the AW surface.

**Figure 6:**
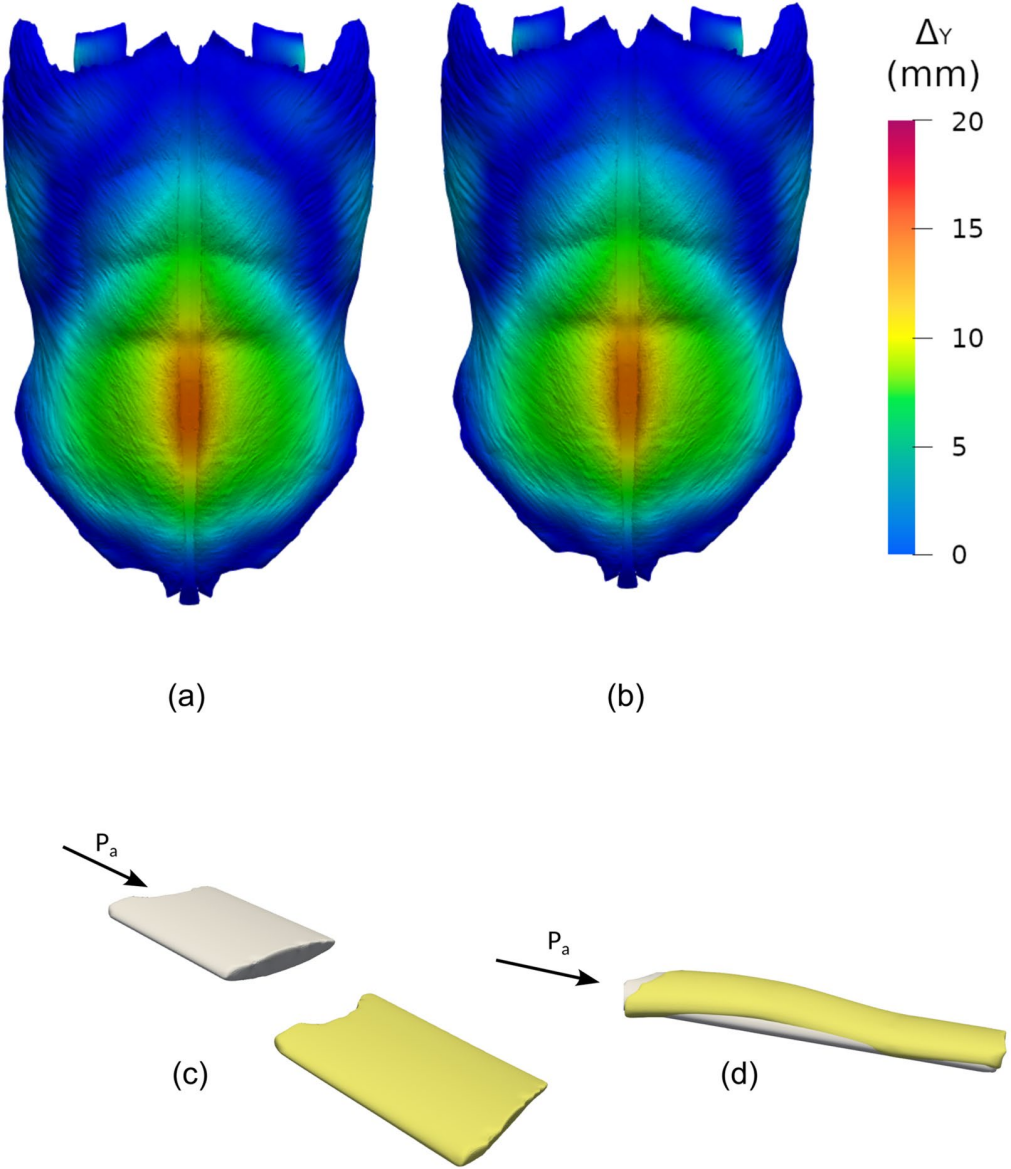
Results from the simulations with *P*_a_ = 20 kPa for soft (E_w_ = 0.1 MPa) A4 (**a**,**c**) and B2 (**b**,**d**) laparoscopy surgical wounds. (**a**,**b**) Deformation of the entire AW external surface is plotted for the simulations with a soft A4 (**a**) and B2 (**b**) surgical wound. (**c**,**d**) Comparison of the original and deformed geometries of A4 (**c**) and B2 (**d**) surgical wounds.

Even though the concept of surface undulation arises intuitively from the visualization of the SW deformed geometry—obtained from the FE simulation output—its quantification is by no means straightforward. In what follows we explain in some detail the methodology that we developed to quantify this concept. Specifically, our main goal is to associate a map of the predicted SW surface changes to a quantity that estimates the SW degree of surface undulation. In the non-deformed geometry, each SW is an elliptic cylinder with two end lids, respectively located on the AW inner and outer surfaces. For every computational node ("i") along the rim of a lid, we determined the nearest node ("j") in the rim of the opposite lid, and we measured the Euclidean distance (d_i,j_) and the minimum geodesic distance (D_i,j_) between these two nodes. A geodesic distance between two points on a surface is the length of a path between the two points along the surface. The full set of distances between rim node pairs was used to compute averaged Euclidean (*d*) and minimum geodesic (*D*) distances for every SW. In the original geometry (Fig. 6a), the SW surface is hardly undulated, and we initially have *D*_0_ ≈ *d*_0_. After a FE simulation was completed, the final Euclidean (*d*_f_) and geodesic (*D*_f_) distances were computed for the deformed SW (see Fig. 6b). The SW undulation was characterized using the averaged tortuosity,

2 $$\overline{\tau } = \frac{{\overline{D}}}{{\overline{d}}}$$

as well as its variation,

3 $$\Delta \tau = 100\left( {\frac{{\overline{\tau }_{f} - \overline{\tau }_{0} }}{{\overline{\tau }_{0} }}} \right)$$

To characterize the compression of the SW due to a decrease in its depth, we defined the averaged geodesic deformation as:

4 $$\overline{G}_{d} = \frac{{\overline{D}_{f} }}{{\overline{D}_{0} }}$$

## Results

Table 4 shows predicted *G*_d_ and $\Delta \overline{\tau }$ values for the simulations with *P*_*a*_ = 20 kPa. Surgical wounds within the LA (A1, A4, B1 and B4) hardly experience any deformation (*G*_*d*_ ≈ 1 and $\Delta \overline{\tau }$ ≈ 0) but they travel a significant distance outward, together with the rest of the LA tissue. Figure 6 shows the predicted deformations for the simulations with *P*_a_ = 20 kPa and either a soft A4 or B2 SW. The deformation behavior of a SW in the LA is illustrated in Fig. 6c for a soft A4 (E_w_ = 0.1 MPa). Laterally located surgical wounds (A2, A3, A5, A6, B2, B3 and B5) hardly change their location instead but their shape is significantly modified (see Table 4). This second behavior is illustrated in Fig. 6d for a soft B2. The depth of laterally placed surgical wounds is reduced, with *G*_d_ values in the 0.96–0.97 range. Also, lateral surgical wounds located in the upper lumbar region (A2, A6 and B2) experience significant surface undulations, as characterized by higher $\Delta \overline{\tau }$ values in Table 4.

**Table 4:** Quantification of SW shape change in simulations with *P*_*a*_ = 20 kPa.

Incision	*E*_*w*_ = 0.1 MPa^a^		Reference case^b^	
	$\overline{G}$_*d*_	∆$\overline{\tau }$ (%)	$\overline{G}$_*d*_	∆$\overline{\tau }$ (%)
A1	1.00	0.00	1.00	0.00
A2	0.97	0.35	0.97	0.30
A3	0.97	0.06	0.97	0.03
A4	1.00	0.01	1.00	0.00
A5	0.97	0.08	0.97	0.05
A6	0.96	0.59	0.97	0.52
B1	1.00	0.00	1.00	0.00
B2	0.97	0.37	0.97	0.30
B3	0.96	0.04	0.97	0.01
B4	1.00	0.02	1.00	0.00
B5	0.96	0.06	0.97	0.01

^a^Just one SW at a time was softened.

^b^In the Reference Case simulation, each incision had the same elastic modulus as the muscles around it.

**Figure 7:**
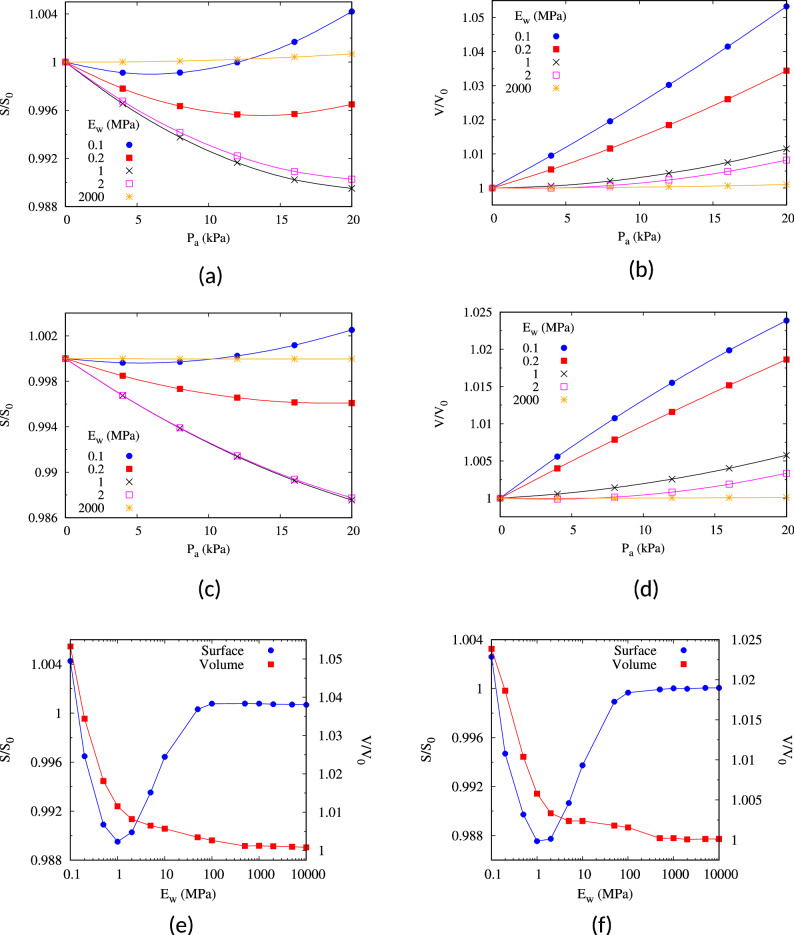
Relative change in surface area (**a**,**c**,**e**) and volume (**b**,**d**,**f**) of the A6 and B2 laparoscopy surgical wounds. In parts (**a–d**), changes in SW geometry are plotted against the level of applied IAP for several values of *E*_w_ whereas in parts (**e,f**) the predicted changes in surface area and volume are plotted against *E*_w_ for the simulations with *P*_a_ = 20 kPa. Calculated values are marked with a symbol whereas the solid line segments are intended just as a visual aid.

To further investigate the mechanical response of the SWs as a function of their toughness (E_w_), we selected one SW from each pattern, namely A6 and B2, which featured the largest surface undulation in terms of *G*_*d*_ and $\Delta \overline{\tau }$ (see Table 4). Figure 7a–d illustrates the relative change of surface area and volume of A6 and B2 as a function of *P*_a_. Figure 7a,c shows that for a very tough SW (E_w_ = 2000 MPa) the surface area of both A6 and B2 is preserved. The surface area of these wounds decreases with increasing IAP, up to about 1% at *P*_*a*_ = 20 kPa, when their stiffness is comparable to that in the surrounding muscles (E_w_ = 1–2 MPa), and it slightly decreases (for P_a_ < 15 kPa) or increases (for *P*_a_ ≥ 15 kPa) when the wounds are soft (E_w_ = 0.1–0.2 MPa). Figure 7b,d shows that the volume of A6 and B2 increases steadily with increasing IAP and with decreasing E_w_, with a maximum expansion of 5.3% for A6 with E_w_ = 0.1 MPa. Figure 7e,f shows that for a fixed IAP level of 20 kPa changes in E_w_ lead to modest changes in SW volume and surface area, with some increase in surface area only observed for the softest SWs (E_w_ = 0.1 MPa). In the 0.2 MPa ≤ E_w_ ≤ 10 MPa range SW surface area decreases instead for both A6 and B2, with a minimum of about E_w_ = 1 MPa.

## Discussion

Even though laparoscopic surgery can be associated with benefits in different procedures when compared with open surgery^20,21^ it might be penalized with an unclear incidence of TSH^1^. TSH incidence turns out to be high when we analyze data from the real-world evidence (i.e., registries)^2^ or we examine medical imaging such as ultrasound and CT scans, which help to diagnose a considerable amount of TSH that would be otherwise clinically undetectable^1^.

Evidence supporting the benefits of trocar-site closure after completing the surgical procedure is very limited. This data uncertainty might be the reason why some authors believe it is not necessary to close the trocar-site wound to prevent a TSH^22^. Nevertheless, the most recent clinical guidelines recommend the closure of trocars with large diameters (10 mm or larger)^4^. In addition to trocar diameter^4^, the location of the trocar-site may affect TSH incidence^22^. Literature reviews suggest higher TSH incidence rates for trocars in the LA, compared to the off midline locations^22^. Moreover, the umbilical trocar site along with the use of larger trocars and obesity have been reported as risk factors for developing TSH^23^. The IAP exerted on the abdominal wall, and consequently on the trocar-site wounds, might also influence the appearance of TSH. Correlation between central obesity and increase in IAP has been described in several works^24–26^.

In the present study, we performed virtual simulations of the mechanical behavior of the AW for different properties of the SW tissues and different IAP levels. Our results indicate that laparoscopic SWs embedded in the LA barely deform as a result of the AW expansion. These wounds, having a diameter smaller than the standard LA width (2 cm), are surrounded by the stiffer LA tissue and thus have very little room for deformation, even when they are very soft (E_w_ = 0.1 MPa). On the contrary, laterally placed laparoscopic SWs significantly deform when the AW is expanded. The volume growth of laterally placed SWs increases with decreasing E_w_, up to a maximum of 5% for E_w_ = 0.1 MPa. A soft SW opposes less resistance to the motion of the muscular tissue (TR and EO) around it and the SW tissue just follows the muscles and adjusts to fill the new space generated. The increase in SW volume is accompanied by either an increase or a decrease in its surface area, depending on the E_w_ value. When a pressure push is applied on the inner AW surface, the innermost muscular layer (TR and EO) is slightly compressed, that is, its depth is slightly reduced. Consequently, the depth of laterally placed SWs is similarly reduced. However, even though SW depth is reduced the increase in SW volume entails an increase in both its length and width. As the total SW surface area is proportional to its depth, length and width, an increase of the two latter quantities can partly compensate, or even outreach, the decrease in the former.

### Study limitations

As a rule, virtual simulations ought to be validated by comparison with experimental results. To the best of our knowledge, no available experimental data exists on the present subject, a fact resulting from the difficulties that are inherent to experimentation with living persons. Nonetheless, the absence of verification constitutes a limitation of the present study.

The present geometrical and mathematical models have several limitations. Only one linea alba geometry, 2 cm wide, has been considered and the present model does not account for the placement of sutures or prostheses at trocar sites. Simulations of surgical wounds located on the LA at the umbilical site level were dismissed. Moreover, a linear isotropic elastic behavior was assumed for all abdominal wall tissues to constrict the computational costs. Even though it is well established that the AW tissues are fibrous and thus anisotropic^27^, our simulations predict modest strain levels even at the highest IAP values considered. That is, proximity to the stress–strain (zero) origin would partly justify the use of the linear constitutive model.

Another limitation of the present study is that only one geometry model, obtained from a specific subject, is considered. This raises a crucial question on the applicability of the present results to the broader population. The substantial inter-subject variability in both morphology and the mechanical properties of the different AW tissues introduces a significant challenge. Therefore, a generalization of the present results would be meaningful at a qualitative level, and even so, it should be approached with caution.

Despite these limitations, we think that the present methodology establishes the basis for future advancements. Specifically, virtual simulations may serve as the basis for a more refined, patient-specific assessment on how trocar placement influences the mechanics of the abdominal wall. Future work should therefore aim to the automation of the geometry building process from individual CT scans, thus offering a more personalized and clinically relevant perspective.

## Conclusion

In conclusion, trocar incisions placed in a LA with non-diastatic dimensions (up to 2 cm) do not seem to compromise the LA mechanical integrity. However, we previously found that trocar holes exceeding the dimensions of a non-diastatic LA can cause an alteration in its mechanical integrity, especially trocars placed in the lowermost area of the LA (hypogastric)^5^. This can be especially important when a surgeon must decide whether to close a trocar placed in the LA, mainly if we consider the “pivot point” or “fulcrum” clinical concept^1^ that affects the part of the trocar held by the abdominal wall muscles around which the trocar performs its movement. Depending on the characteristics of the intervention, the trocar will pivot around a distinct set point, potentially causing shearing injury to the abdominal wall, enlarging the defect in the abdominal wall musculature. Maybe robotic trocars, which move with more precision, would maintain the integrity of the LA without exceeding its dimensions because they are pivoting in the same set point all time, regardless of the characteristics of the intervention. Laterally placed trocar incisions (beyond the rectus abdominis muscles) experience greater deformation with IAP than those placed on LA. The more lateral the trocars are placed the greater is their deformation, regardless of their size. It could be speculated that the mechanical behavior of lateral trocars may be compromised if left only at the expense of scar resistance, with potential risk of finally developing TSH. It might be, therefore, advisable to close lateral trocars with a suture or even use a prosthetic reinforcement depending on the patient's risk factors (e.g., obesity) and regardless of the size of the trocar.

## Data Availability

The datasets used and/or analyzed during the current study are available from the corresponding author on reasonable request.
